# Rapid Ethical Appraisal: A tool to design a contextualized consent process for a genetic study of podoconiosis in Ethiopia

**DOI:** 10.12688/wellcomeopenres.12613.1

**Published:** 2017-10-10

**Authors:** Tewodros Tariku Gebresilase, Zebene Deresse, Girmay Tsegay, Tesfaye Sisay Tessema, Abraham Aseffa, Gail Davey, Melanie Newport, Fasil Tekola-Ayele, Adamu Addissie

**Affiliations:** 1 Armauer Hansen Research Institute, Addis Ababa, Ethiopia; 2 Unit of Health Biotechnology, Institute of Biotechnology, College of Natural and Computational Sciences, Addis Ababa University, Addis Ababa, Ethiopia; 3 Debre Markos University, Debre Markos, Ethiopia; 4 The Wellcome Trust Brighton and Sussex Centre for Global Health Research, Brighton and Sussex Medical School, Brighton, BN1 9PX, UK; 5 Centre for Research on Genomics and Global Health, National Human Genome Research Institute, National Institutes of Health, Bethesda, MD, 20892, USA; 6 Medical Faculty, Addis Ababa University, Addis Ababa, Ethiopia

**Keywords:** Informed consent, Rapid Ethical Appraisal, Podoconiosis, Developing Country

## Abstract

**Background:** Obtaining genuine informed consent from research participants in developing countries can be difficult, partly due to poor knowledge about research process and research ethics. The situation is complicated when conducting genomic research on a disease considered familial and a reason for stigmatisation.

**Methods:** We used a Rapid Ethical Appraisal tool to assess local factors that were barriers to getting genuine informed consent prior to conducting a genetic study of podoconiosis (non-filarial elephantiasis) in two Zones of Ethiopia. The tool included in-depth interviews and focus group discussions with patients, healthy community members, field workers, researchers/Institutional Review Board (IRB) members, elders, religious leaders, and podoconiosis administrators who work closely with patients.

**Results:** Most patients and healthy community members did not differentiate research from routine clinical diagnosis. Participants felt comfortable when approached in the presence of trusted community members. Field workers and podoconiosis administrators preferred verbal consent, whereas the majority of patients and healthy community members prefer both verbal and written consent. Participants better understood genetic susceptibility concepts when analogies drawn from their day-to-day experience were used. The type of biological sample sought and gender were the two most important factors affecting the recruitment process. Most researchers and IRB members indicated that reporting incidental findings to participants is not a priority in an Ethiopian context.

**Conclusions:** Understanding the concerns of local people in areas where research is to be conducted facilitates the design of contextualized consent processes appropriate for all parties and will ultimately result in getting genuine consent.

## Introduction

Informed consent is a prerequisite for conducting ethical research on human participants. It is considered valid when participants (i) receive appropriate and comprehensive information, (ii) understand essential aspects of the research, (iii) voluntarily participate without any form of coercion, and finally (iv) agree to participate by giving either written or verbal consent.

Even though these principles are endorsed by many international and national ethics guidelines, the reality is far from this, and there is still a gap in achieving genuine informed consent, especially in studies conducted in developing countries. A meta-analysis of 21 studies conducted in Africa indicated that comprehension of key concepts (for example, randomization and placebo) differed significantly among participants, and “therapeutic misconception” is a very common issue ^1^. Therapeutic misconception occurs when participants do not understand the distinction between research and clinical care, and believe the purpose of their participation is to get some form of treatment rather than to generate new data. Some of the reasons for such misconceptions are high disease burden, poor access to health care, and low literacy levels in these countries ^1^. Another issue affecting the consent process is the extent to which a community or family interferes with the autonomous decision-making capacity of an individual. Western ethics guidelines place a huge emphasis on autonomous decision-making, and this may be contrary to the decision-making practice in most rural African settings ^2, 3^.

The situation is further complicated in obtaining informed consent for genomic research due to the broad and unexpected nature of results generated from such studies. For example, in conducting sequencing and genotyping studies, researchers might discover genetic variants, which increase the risk of developing certain diseases. Such findings are medically relevant and are termed as ‘incidental findings’. In Africa, the rapid decline in sequencing cost and advances in technologies has prompted scientists to undertake genomic studies, and it is essential to develop guidelines on how to manage incidental findings in order to conduct ethical research.

In the past few years, many studies have promoted the need to contextualize the informed consent process to develop one that is ethically sound and culturally sensitive ^4, 5^. One tool recently employed to explore barriers to ethical conduct of research and tailor the consent process to the local context is Rapid Ethical Appraisal (REA) ^2, 3, 6– 8^. Similar to traditional qualitative studies, REA use in-depth interviews (IDIs), focus-group discussions (FGDs) and observation to collect data from key community informants. However, it is faster and more cost-effective in generating insights and reconciling western ethical standards with the context of developing world research settings ^6, 9^.

The validity and feasibility of REA has been assessed recently in Ethiopia and was found to be relevant and acceptable in exploring social and cultural issues affecting the ethical conduct of research ^9, 10^. Using this tool, for example, Tekola *et al.*, found that podoconiosis patients in Wolaita, Southern Ethiopia, were afraid to participate in a genetic study for fear that the study might confirm the hereditary nature of the disease and fuel the existing social stigma ^11^. Studies in Cameroon also identified a number of issues regarding how to approach and obtain informed consent from participants involved in podoconiosis genetic research ^2, 7^.

Even though the tool has been employed previously to explore ethical issues in Ethiopia ^3, 11^, issues identified in Wolaita might not be applicable to other Ethiopian populations that differ in their social and community structure. The purpose of this REA study was therefore to explore barriers to getting genuine informed consent prior to enrolling participants in the genome-wide association study (GWAS) of podoconiosis in East Gojjam and East Wellega Zones of Ethiopia.

## Methods

### Ethical consideration

Ethical approval was obtained from the Armauer Hansen Research Institute (AHRI)/All Africa Leprosy and Tuberculosis Rehabilitation and Training Centre (ALERT) Ethics Review Committee, Addis Ababa, Ethiopia (registration number PO20/12) and the National Research Ethics Review Committee, Addis Ababa, Ethiopia (reference number 3.10/577/06) before conducting the study. Permission was also obtained from the Oromia and Amhara regional health bureaus. We obtained verbal consent instead of written consent as we were exploring the preferred method of consent documentation in our study area.

### Study area and settings

**Figure 1.**
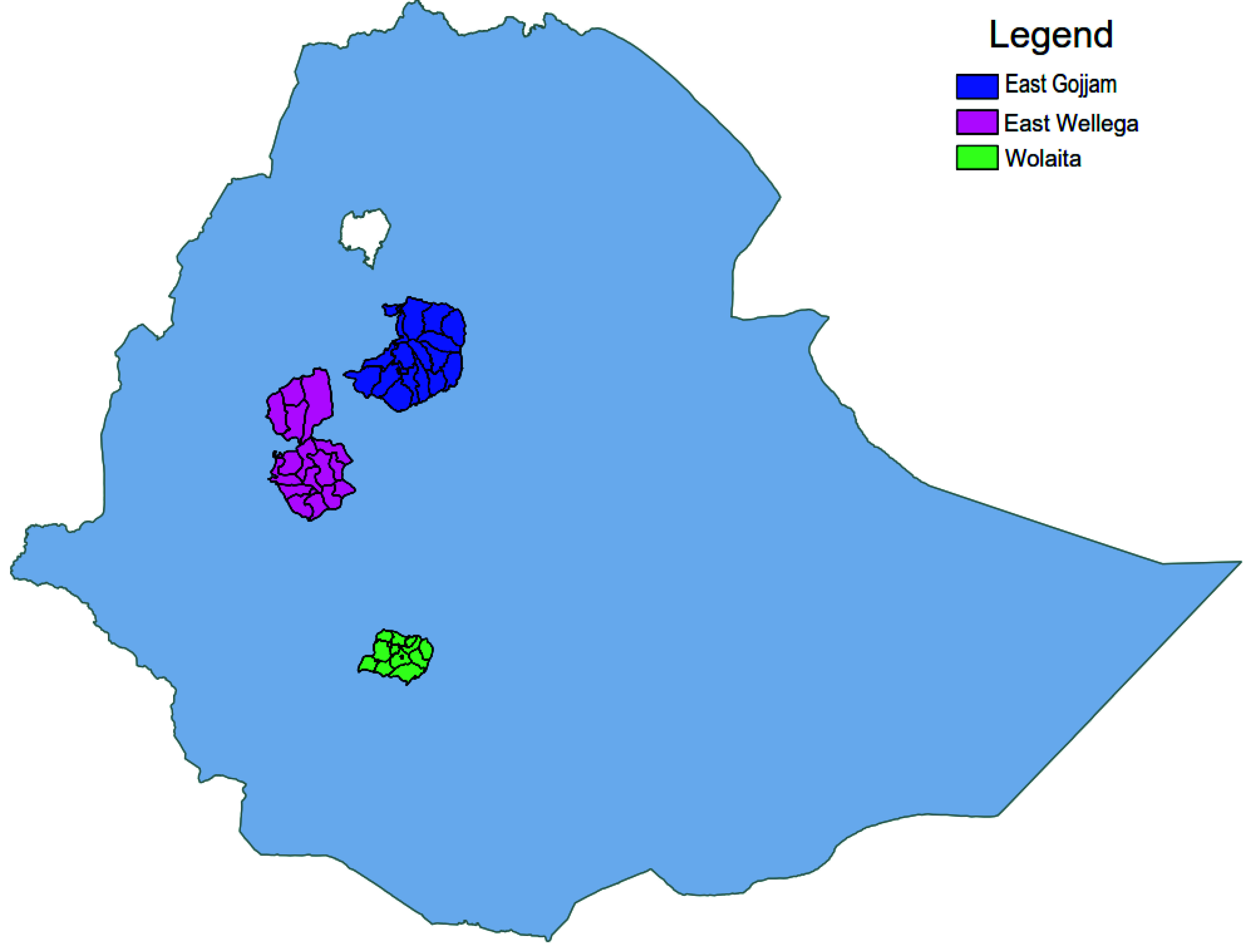
Map of Ethiopia showing the two study areas (East Gojjam and East Wellega). Wolaita, where the first Rapid Ethical Appraisal study was conducted, is shown for reference.

This study was conducted from February to April 2014 in East Gojjam and East Wellega Zones ( Figure 1) of North and North-Western Ethiopia, respectively, prior to enrolling participants to the podoconiosis genetic association study. This study aimed to validate the association between podoconiosis and genetic variants in the HLA region using DNA from saliva sample ^12^ in Wolaita, Amhara and Oromo ethnic groups in Ethiopia. Amharic and Afaan Oromo are the most widely spoken languages in East Gojjam and East Wellega (99% and 88% of the population, respectively). The majority of the population in these two Zones are subsistence farmers living in rural areas (92.28% in East Wellega and 90.08% in East Gojjam). The prevalence of podoconiosis among individuals aged 15 years and above was reported to be 3.3% in Gojjam ^13^ and 2.8% in Wellega ^14^.

### Study design and participants

We conducted IDIs and FGDs with podoconiosis patients, healthy community members, podoconiosis administrators, elders/religious leaders, field workers, *kebele* (the smallest administrative unit in Ethiopia) leaders, and researcher/IRB members to explore their views on the consent process. The FGDs were comprised of single-sex adults each containing 5 to 7 individuals. Participants were chosen purposively based on their ability to discuss the issues openly and inform the phenomenon under investigation. Participants were chosen in consultation with field workers of the International Orthodox Christian Charities (Debre Markos, Gojjam) and the Ethiopian Catholic Church Clinics (Nekemte, Wellega). The field workers are experienced (>3 years of experience working in the community) and provide treatment and rehabilitation services to the communities affected by the disease. Recruitment and interviewing continued until information saturation was reached and no further ideas were generated from additional interviews.

### Data collection

Guide questions were prepared for each interviewee group in English ( Supplementary File 1), and were then translated into Amharic and Afaan Oromo by an independent person. The interviews were back-translated by an investigator (ZA) who was not involved in conducting the interviews. With the exception of three interviews conducted in English (with podoconiosis administrators), all other interviews in East Wellega were conducted in Afaan Oromo. All the interviews in East Gojjam were conducted in Amharic. The interviews were conducted by the principal investigator (TTG) and the two co-investigators (FTA, GT), who had experience in conducting qualitative research.

### Data analysis

The audio of the interviews were transcribed and translated to English by the principal investigator and two co-investigators, and checked for inconsistencies. Transcripts were imported to NVivo 10 software (QSR International) and coded independently by the PI and the co-investigators. Thematic analysis was then used to identify patterns and themes using a conceptual framework described in previous studies ^2, 3, 7, 11^.

## Results

In total, 43 IDIs and 5 FGDs were conducted ( Table 1) with 31 men and 20 women from East Gojjam, and 16 men and 8 women from East Wellega. The age of respondents ranged from 18 to 68.

**Table 1.** Type and number of interviewees in each group. IDI, in-depth interviews; FGD, focus group discussion.

Participant type	East Gojjam		East Wellega	
	IDIs	FGDs	IDIs	FGDs
Patients	8	1 (n=6)	5	-
Healthy Community Members	1	3 (each n=5, 6, 7)	2	1 (n=7)
Podoconiosis administrators	2	-	3	-
Elders/religious leaders	4	-	2	-
Field Workers	3	-	3	-
Kebele Leaders	2	-	2	-
Researcher/IRB members	7 IDIs			

The issues that arose from the analysis were grouped into the following themes: perceptions towards health research and informed consent, preparation of information sheet and consent form, approaching the community, information provision, assessing comprehension, decision-making, recruitment, consent taking, and return of incidental findings.

### Perceptions on health research

The majority of healthy community members and patients in the rural areas had incorrect or no understanding about research. They described research as a routine health care activity with the goal of providing treatment for their illness. Few could correctly distinguish between the two concepts.

*“I believe research is a tool to maintain our health. We know our health status after participating in a research.” (Patient 9, Gojjam).*

*“I don’t have any idea about this [research].” (Patient 2, Wellega).*

*“Research is used to increase new knowledge, whereas diagnosis is to find the disease.” (Patient 7, Gojjam).*

Some participants’ responses implied that research involving questionnaire-based data collection might be confused with aid eligibility assessment or criminal investigation.

*“Nobody knows what it is, but they [the community] recognize, if new face comes with questionnaire, whether that is short or long, something will come after a while. That is their expectation.” (Podoconiosis administrator 2, Wellega)*.

The misconception regarding research and criminal investigation/diagnosis arise from the use of Amharic words that sound the same but have different meanings - a term referred as homophones.

*“As a result of their past political and social experiences, the society differ in their understanding of words that are used to describe research, and care must be taken in this regard. For example, they may associate research with crime, police investigation and being a witness for someone.” (Podoconiosis administrator 2, Gojjam).*

When asked to participate in research (in Amharic “ *Meremer*”), some participants confused this term with another Amharic term “ *Mermera*” which can imply either clinical diagnosis or police investigation. Moreover, research-involving drawing blood may be mistaken for screening for diseases, particularly HIV/AIDS.

*“When you tell them that you are doing research, all people think it is HIV testing. It is not clear to them. They take research and diagnosis as the same.” (Elders/religious leaders 3, Gojjam).*

*“Research is used for identification of diseases during illness and for checking oneself (HIV-testing). People go to health facilities to get tested for HIV.” (Elders/religious leaders 1, Wellega).*

### Perceptions on informed consent for health research

There was unanimous agreement among all researcher/IRB members and field workers interviewed that informed consent is a tool to communicate the study to potential participants and is essential to maintain quality of research.

*“Many research participants could be vulnerable due to lack of knowledge; even educated people can sometimes be vulnerable. So, the information sheet is a tool to describe the basic of the study in an easy language.” (Researcher/IRB member 2).*

*“People usually give genuine answer when they are relaxed and free. They might not give you genuine answer if they are forced or coerced.” (Researcher/IRB member 6).*

*“Informed consent is a process of obtaining permission from our participants.” (Researcher/IRB member 1).*

### Preparation of consent form and information sheet

Participants stressed the need to express study information in a simple and precise manner using few technical terms.

*“Some researchers don’t know what kind of information they provide. They put everything research participants do not necessarily have to know. I received 20-page information sheet and consent form when I worked as ethics secretariat. It is amazing. It shows lack of confidence” (Researcher/IRB member 4).*

Patients and healthy community members wanted to be told about the objectives, implications and benefits of the study, as well as cause and prevention of podoconiosis before deciding to participate in the research. On the other hand, researcher/IRB members insisted on a need to inform prospective participants the type of study, foreseeable risks, confidentiality, voluntariness, and contact details of the principal investigator (s) and ethics committee(s) that approved the study. 

*“Healthy people will be volunteer if you tell them first about the cause of the disease and then what you will do to their saliva sample.” (Healthy community member 3, Gojjam).*

*“I want to be told about the importance of the study to my health.” (Podoconiosis patient 3, Wellega).*

*“Confidentiality should be secured but it is a very difficult issue since most often many people are around…You may make an effort to keep people out of the hearing distance but in a village setting it is often not easy. Then the idea of confidentiality may also be a bit different because people have most of their private things in public. Is it not?" So, I think that is the most important thing, confidentiality.” (Researcher/IRB member 3).*

Some researcher/IRB members suggested doing pre-testing of the consent form and information sheet in the study site to assess its suitability and appropriateness.

*“If participants are from rural areas, the information sheet and consent form have to be pretested in advance. Then we can use it if they say it is appropriate. Sometimes when I ask participants, they ask me back questions saying, “What do you mean by that?” So, sometimes you can learn words or phrases from participants that you can use to describe certain information.” (Researcher/IRB member 5).*

### Approaching potential study participants

Many participants suggested approaching participants in the presence of known and trusted community members, including religious and *kebele* leaders, health extension workers, patient association leaders, local NGO staff. Others also mentioned using existing governmental or social structures, such as the ‘1-to-5' networking scheme (a government-created structure where one member networks with five other people and leads the group to discuss societal issues) and *Idir* (locally established traditional self-help financial associations).

*“There is a local saying “Yeagerun bere beageru serdo”, which is to mean “local problems can be best solved by the local people.” …They might be suspicious if you read the information to them, but they might not if someone they know reads it in front of them. You can ask the kebele administrators to read the consent form in front of them.” (Field worker 3, Gojjam).*

*“… They prefer to see someone whose face is not new to them.” (Podoconiosis administrator 2, Wellega).*

*“The health extension workers are better since it is related to health. The people may assume that they are summoned for a government meeting if they are called by the kebele administrator.” (Healthy community member 2, Gojjam).*

*“If you want the community at large, Kebele is better. If small group is needed, we [the church] can call them.” (Podoconiosis administrator 1, Wellega).*

There was considerable discussion for and against the involvement of health extension workers (HEWs) in approaching community members for research. Most participants said that *kebele* leaders are important to confirm credibility of the study and approach prospective participants, whereas HEWs would be more appropriate to discuss health-related issues. Some participants, however, cautioned against employing HEWs because of their already-stretched work schedules.

It was indicated that the best time to approach potential participants is during the dry season, weekends (preferably Sunday) or holidays (especially religious holidays) when they are not actively engaged in farming activities. 

*“You can recruit many people as long as your schedule coincides with participants. You have to be very flexible. You may find a religious leader working as a farmer, so you can interview him under the shade of a tree.” (Researcher/IRB member 3).*

### Information provision

The local people use the Amharic term “ *Zer*” or “ *Zer kotero meta*” to describe hereditary diseases passed across family. Field workers and podoconiosis administrators warned that such concepts must be explained cautiously so participants do not consider podoconiosis as a hereditary disease.

*“It is difficult to tell podoconiosis susceptibility concept. We do not tell them directly. If we do, they may consider susceptibility as a hereditary condition, and this may affect our work.” (Podoconiosis administrator 2, Gojjam).*

*“…Our target was not to put the genetic component of the disease at the centre of their attention. The community worried and think it [podoconiosis] is inherited. As I told you before, we try to give them examples and show them that they get the disease from the soil and by not wearing proper shoes…So, if somebody comes and talks about hereditary nature of podoconiosis, it counteracts our efforts. So can’t you put it in a broader sense like various ideas where this disease can come from?” (Podoconiosis administrator 3, Wellega).*

Participants better understood genetic variation and difference in disease susceptibility concepts when analogies drawn from their day-to-day observations were used. In particularly, many participants were familiar with the notion of improved seed yielding bigger crops, which proved to be a useful analogy. Podoconiosis administrators also mentioned that they pose a question to participants “why do some people get sick with the common cold and some do not despite living in one household?” to explain susceptibility difference in a family. They said a two-way conversation and probing is helpful to promote understanding of abstract concepts.

*“...We tell them that all their family members do not develop common cold at once; some members may and some may not. So you can explain the concept of genetic susceptibility this way.” (Podoconiosis administrator 2, Gojjam).*

*“I don’t know how to explain this. It is difficult to me. Maybe a good explanation is needed...focusing on the nature of foot sole thickness and how the diseases go through family could also help.” (Field worker 2, Wellega).*

### Assessing comprehension

Field workers and podoconiosis administrators suggested to assess comprehension by asking open-ended question and said that this approach can clear any potential misconceptions about the study and enhance participants’ understanding of key study information.

*“After you provide information about your study, ask them to tell you what they understood from your talk. That way you can assess their understanding level.” (Field Worker 1, Gojjam).*

*“Once you told them about the study in their local language, ask them open-ended questions to check if they understand what you mean.” (Podoconiosis administrator 1, Gojjam).*

*“After you explain the study, ask 3 or 4 participants to explain to others what you have been saying. This can help others who have misunderstood your explanation to catch-up with the group.” (Field Worker 2, Gojjam).*

### Decision-making, recruitment and consent taking

Most participants said that they could decide by themselves as long as they are not asked to come repeatedly, cover their own costs, stay away from their home for a long time, or are required to use family planning methods (a decision that is usually made after discussing with their partners). Some also said they reach a decision on their own if the research benefits their health – further evidence that ‘therapeutic misconception’ is common in society.

*“The problem is if I am asked to pay a certain fee. So it is better if I decide together with my partner.” (Patient 2, Gojjam).*

*“I don’t think they will discuss with their family. The only time they do that is when they are asked to use family planning methods.” (Kebele leader 2, Gojjam).*

*“It depends on the decision to be made. They will definitely discuss with their family members if, for example, the treatment site is far and are asked to stay for 15 days or spend 7 days for foot massage. The whole family discuss and debate whether that person should go or not, and at the end, the idea accepted by most people win.” (Podoconiosis administrator 2, Gojjam).*

Several factors that could potentially influence the recruitment process were raised and these are listed in Table 2.

**Table 2.** Factors affecting recruitment to research.

*Factors*	*Description*
Gender	*Some females (especially those that are married) might not be comfortable* *being interviewed alone with a stranger.*
Sample type sough	*Saliva is preferred over blood and other samples that require invasive collection* *procedures. Some associate blood sampling with HIV testing and sorcery.*
Perception towards local government officials	*Individuals who are not happy with local government officials may decline to* *participate in a research.*
Previous exposure to research	*Individuals with previous research exposure were not suspicious that their* *samples will be used in sorcery activities.*
Economic status	*Some people who are economically better might be offended when they are* *offered incentives, whereas those who are not economically strong may decline* *to participate for fear that they will be charged fees.*

We found mixed views regarding the preferred form of consent documentation. Field workers and podoconiosis administrators preferred verbal consent, whereas the majority of patients and healthy community members suggested the use of both verbal and written consent to accommodate the needs of literate and illiterate participants.

*“First, they have to be educated. They sign a consent form freely if they understand it well. If they are asked to sign without explanation, they may think something bad is done behind them.” (Healthy community member 4, Gojjam).*

*“They think something tangible if it is written and read to them. There is a local saying that” what is written on paper and what a fool once understands can never be lost or forgotten!” So, they want written information.” (Field Worker 2, Wellega).*

*“Healthy people might be afraid of signing; they might associate it with bad experience such as tax or confiscating their property. The situation can even be difficult with illiterates. They are suspicious and might think what is written on the document and what is read for them is different.” (Field Worker 1, Gojjam).*

### Return of Incidental findings

We finally asked researcher/IRB members to share their views about returning incidental findings in an Ethiopian context. Incidental findings are medically relevant information encountered in the course of a study but are beyond the aim of the study for which participants originally consented. The majority of researcher/IRB members were against disclosing incidental findings and felt that “not knowing is better than knowing”, given the poor health system in low-income countries.

*“There are many severe and disabling conditions, and up to now, the health structure hasn’t been very good in addressing these. I find it unethical to tell participants they have susceptibility to rare cancer, which has no cure.” (Researcher/IRB member 6).*

*“In a country where we even don’t have enough drugs for headache, the disadvantage to know incidental finding weigh more than its advantage.” (Researcher/IRB member 5).*

A few, however, said that the issue should be decided by stakeholders, including the IRB and family members. They all agreed, however, to provide participants the option whether they would like to receive their incidental finding result, but were sceptical if participants, especially those living in rural areas, understand the implications of their decision.

*“I don’t think it is appropriate and ethical to reveal the result [incidental findings] unless the patient expresses their consent.” (Researcher/IRB member 4).*

*“The main difficulty that you will face is explaining incidental finding to participants.” (Researcher/IRB member 3).*

## Discussion

Obtaining genuine informed consent from participants in low-income countries can be difficult, partly due to poor knowledge about research processes and research ethics ^15^. Cultural and social values surrounding disease and illness and familiarity with evidence-based research are among the many factors that influence the consent process ^16^. Using REA as a tool, we identified a range of factors that can act as barriers to gaining genuine consent. We subsequently used these findings to design a contextualized consent process for a genomic study of podoconiosis (study completed; manuscript currently under preparation). 

Most participants residing in rural areas did not know the distinction between research and health care and thought they were being screened to receive treatment rather than participate in research. Therapeutic misconception is common in low-income countries ^1, 3, 7, 17, 18^, especially in clinical research where providing treatment and conducting research are often carried out simultaneously ^19^. Researchers conducting studies in such settings must assess participants’ motivations for taking part in the research. In our main genetic study, we explicitly informed participants that we were not affiliated with the local NGOs, which provide podoconiosis treatment services to the communities, and employees of the NGOs who helped us facilitate participant recruitment. 

Similar to findings of Tekola *et al*. ^3^, trusted individuals are the preferred entry point to the community. Community engagement and sensitization is critical to gain access, build trust and provide study-related information to prospective participants. For example, a religious leader spread a rumour that the vaccine used for polio immunization was contaminated with anti-fertility substances. This negatively impacted on uptake rates and resulted in discontinuation of the vaccination campaign ^20^. The campaign was later resumed after discussion with the religious leaders and convincing the population that the vaccine was safe and protects people from polio. This example highlights the influential role of community leaders and the importance of engaging them when conducting community-based studies or interventions.

Our study indicated that prospective participants need to know basic information about the study and did not require detailed information to take part in our study. Even though most information sheets and consent forms contain the purpose and objective of the study, it is important to cover all aspect of the research to help participants reach an informed decision. In the context of genomic studies, this includes information about the nature of the study, privacy, confidentiality, future use of samples and data/sample sharing plan. Participants in rural settings might not fully understand some of these concepts and a thorough explanation is needed to create awareness. In a study conducted in rural Ghana, for example, participants did not completely understand a statement about future use of stored samples, but grasped the idea when the concept was broadly explained using familiar examples, such as that “their left-over samples could be used in future studies when new ideas come up” ^21^. Similarly, explaining hereditary concepts and maintaining a balance with transparency was challenging but could be done if the community’s understanding is assessed prior to conducting the study.

In our study, we explained genetic susceptibility concepts using the common cold analogy described above, saying that individuals from the same family differ in their ability to fight infections. Thus, simple and real-world examples must be used to describe abstract and scientific concepts so that participants can fully comprehend the nature of the study in which they agree to participate ^18, 22^.

The field workers suggested that probing and checking the level of understanding of key study information in a question format (rather than using the traditional binary form of assessment, e.g., yes/no format) could improve comprehension. Lindegger *et al.* used 4 tools (self-report, checklist, vignettes, and narrative measures) to assess comprehension, and higher scores were recorded when checklists were used, indicating that the level of understanding is dependent on the type of tools used ^23^. The method suggested by the field workers needs further evaluation to check its appropriateness to the local settings. We did not implement this suggestion in our work because of resource constraints.

Participants also indicated that the language used to describe the research process could affect the consent process. For example, some may take the Amharic translation for the English word research “Mermer” to mean a police/criminal investigation. Words can have different meaning depending on the context and settings ^24^, and to avoid confusion, it is recommended to pre-test the information sheet and consent form to evaluate its appropriateness in the target population. In our case, we avoided using “ *Mermer*” when describing research and used unambiguous words (e.g., “ *Tinat*” for research) that reduced the possibility of negative interpretation by the community. Such an approach has been suggested by Molyneux *et al.*, who studied informed consent processes in Kenya ^25^.

Participants had misconceptions linking research with aid and expected monetary or in-kind benefits. Even though acceptable compensation is appropriate, any available benefits in resource-limited settings might result in undue influence on participants ^26, 27^. We discussed this issue with field workers and podoconiosis administrators and they suggested providing soap and other sanitary materials to promote foot hygiene, as an alternative to monetary compensation. The issue of ‘appropriate’ compensation is often controversial and context-specific, and to this end, local regulatory authorities (including ethics committees) must work with the community and researchers to establish reasonable compensation system.

Signing a consent form was found to be acceptable to patients and healthy community members and both groups supported the use of either written or verbal consent. We chose to document verbal consent for the REA study, whereas written consent was used in the main genetic study since institutional and national ethics committees in Ethiopia require written consent when conducting genetic studies on humans.

Many factors that could potentially affect participants’ recruitment were identified. Among these, gender and type of sample sought were considered by field workers and podoconiosis administrators to be the most important. In some communities, cultural practices negatively influence women’s participation in some types of research studies (e.g. sexual health issues) ^28^, and permission might be required from their husband to participate in such studies. A study conducted in Qatar, for example, indicated that a majority of Muslim female participants felt they should not be interviewed in a private room with a man and preferred the outpatient waiting area where they can be seen in public ^29^. In our genetic study, enrolment was carried out in public places (clinics, schools and *kebele* compounds) and questions that can be considered sensitive were not asked. If gender issues are anticipated, however, researchers are advised to assess the existing gender norms in their proposed study area and design culturally sensitive ways to approach potential participants (e.g., gender-matching between researchers and participants) ^28^.

All participants preferred to give saliva compared to other sample types that require invasive collection procedures (e.g., blood). Blood sampling is a very sensitive issue in most rural African settings ^25^. In a study conducted in Nigeria, for example, participants complained their blood could be used in sorcery activities ^30^. On the contrary, participants in our settings did not associate saliva with sorcery; some were even surprised that their saliva sample could be helpful for research or laboratory-based diagnosis.

In the past few years, the cost of genome sequencing has plummeted dramatically, and this has resulted in an increasing number of genomic studies. The ethical, legal and social implications (ELSI) of such studies are extensively considered in Western countries ^31^, but they are not given much emphasis in Ethiopia as the medical application of genetics (e.g., genetic counselling, pharmacogenetics, etc.) is not widespread in the country. However, Ethiopian scientists are involved in national and international collaborative genomic studies (e.g., Human Heredity and Health in Africa project) and a consensus must be reached on how to handle the ELSI issues. In our study, we particularly raised the issue of incidental findings to researcher/IRB members and the majority of them were against disclosing incidental findings arguing that there are other health issues (e.g., providing child-maternal health care) to which priority must be given. In their review, Wright *et al.*, discussed the ethical issues in conducting large-scale genomic studies in African population ^32^, and they indicated that disclosing incidental findings is a complex issue in most countries. For example, whose duty is to inform incidental findings to research participants? Which results must be returned? Some of these issues are philosophical and active debate must continue between various stakeholders of the research enterprise to come up with a recommendation for the Ethiopian context.

## Conclusions

Our study indicated that a ‘one-size fits all’ approach does not work when it comes to consent process. As described in Addissie *et al.* (2015), REA might not be required for all studies and the decision to employ the tool should be based on many factors, including the community where the study is to be conducted, the research topic, and the availability of resources ^9^. However, REA is especially useful to explore issues in studies where ethical dilemmas are anticipated, including randomized clinical trials, studies conducted in research-naïve areas, and research involving sensitive topics and vulnerable groups ^33^. Understanding local issues will help to design contextualized consent processes appropriate for all parties and ultimately in getting genuine consent.

## Data availability

The transcripts from all IDIs and FGDs are available from OSF: http://dx.doi.org/10.17605/OSF.IO/AWKD4 ^34^. These transcripts are de-identified to maintain appropriate levels of anonymity.

## Supplementary material

Supplementary File 1: Guide questions for the interview with different stakeholders.

Click here to access the data.
