# Supplementary material for: Rapid Ethical Appraisal: A tool to design a contextualized consent process for a genetic study of podoconiosis in Ethiopia
Source: Wellcome Open Res. 2026 Mar 10;2:99. Originally published 2017 Oct 10. [Version 2] doi: 10.12688/wellcomeopenres.12613.2 (PMC13010092; doi:10.12688/wellcomeopenres.12613.2)
Supplement: Peer review history

# Reviewer report: Reviewer response for version 2

**DOI:** 10.21956/wellcomeopenres.28627.r150536

**Published:** 2026-04-08

**Authors:** Signe Mežinska

**Affiliations:** 1 University of Latvia, Riga, Latvia

This is an interesting study, and I thank you for the opportunity to review the revised version. I have the benefit of having read the other reviewers’ reports and the authors’ responses, and I therefore limit my comments to an overall assessment of the revised manuscript and whether the key concerns previously raised have been adequately addressed.

 In general, the authors have responded to the concerns raised in the initial round of peer review. The revised manuscript shows a much improved alignment between introduction, results and discussion. The introduction now provides a broader discussion on the ethical challenges associated with genomic research beyond incidental findings, better reflecting the scope of results and discussion. The initial confusion between consent as a process and written documentation has been solved.  Methods are described in more details, following the reviewers’ comments. Also, the discussion is reorganized, and limitations section is strongly amended to accommodate peer reviewers’ comments. Nevertheless, there are some unresolved issues:

- The authors have implemented the suggestions to change or clarify terminology (e.g., developing countries vs. LMIC, genuine informed consent vs. valid informed consent); however in some cases the changes are not fully implemented:
  - there are still two mentions of “genuine informed consent” in the introduction;
  - the authors write “We have updated the terminology from "Podoconiosis administrators" to "care coordinators" throughout the manuscript to better reflect their role”, nevertheless there are still several mentions of "Podoconiosis administrators" throughout the manuscript;
- The authors have followed the reviewer’s suggestion to correct the inconsistent verb tense, nevertheless, in the results part there are some sentences where present tense still appears.

The data analysis in the second version of the article is richer and more interpretative than in the first version, but it remains moderately descriptive rather than deeply analytic. The results section relies heavily on interview and focus group quotations, which illustrate participants' views, but the commentaries are often brief and explanatory.  Clearer integration of analytic insight withing the results would strengthen the analysis. There are also still some statements in the discussion where the authors move from descriptive findings to normative recommendations (e.g., regarding how consent processes should be structured or what constitutes an adequate information) without always making explicit how far these recommendations are grounded directly in data versus broader ethical reasoning. It would be advisable to clearly distinguish whether normative claims are based on data or broader ethical literature.

 The conclusions part moves quickly to generally endorsing the value of contextualized consent and REA , without sufficiently summarising the empirical barriers identified in the study (the aim of the study was "to explore barriers to getting genuine informed consent prior to enrolling participants in the genome-wide association study”. As a result, conclusions somewhat overgeneralize beyond the aim of the study.

 Some minor grammatical issues:

1. Hyphenation for “low-income” is not consistent (there are different forms used – lowincome, low income and low-income).
2. In two sentences there is a misspelling of LMIC  (LIMC): “However, it is faster and more cost-effective (typically taking 4–6 weeks and costing ~2,250 USD per site) in generating insights and reconciling western ethical standards with the context of LIMC research settings.”  and “Therapeutic misconception is common in LIMC”.
3. In the results section, most of the text is in the past tense, but occasional present-tense constructions appear. This would require copy editing.

In general, this is a manuscript that makes a valuable contribution to discussions on informed consent in low‑resource settings, and with minor further refinement it will be well placed for indexing.

Is the work clearly and accurately presented and does it cite the current literature?

Yes

If applicable, is the statistical analysis and its interpretation appropriate?

Not applicable

Are all the source data underlying the results available to ensure full reproducibility?

Yes

Is the study design appropriate and is the work technically sound?

Yes

Are the conclusions drawn adequately supported by the results?

Partly

Are sufficient details of methods and analysis provided to allow replication by others?

Yes

Reviewer Expertise:

Biomedical research ethics, bioethics, medical ethics, sociology of health and illness.

I confirm that I have read this submission and believe that I have an appropriate level of expertise to confirm that it is of an acceptable scientific standard, however I have significant reservations, as outlined above.

# Reviewer report: Reviewer response for version 2

**DOI:** 10.21956/wellcomeopenres.28627.r150331

**Published:** 2026-03-23

**Authors:** Jantina de Vries, Syntia Nchangwi Munung

**Affiliations:** 1 Department of Medicine, Faculty of Health Sciences, University of Cape Town, Cape Town, South Africa

I am satisfied that the authors have addressed all questions that we raised in our review.

Is the work clearly and accurately presented and does it cite the current literature?

Yes

If applicable, is the statistical analysis and its interpretation appropriate?

Not applicable

Are all the source data underlying the results available to ensure full reproducibility?

Partly

Is the study design appropriate and is the work technically sound?

Yes

Are the conclusions drawn adequately supported by the results?

Partly

Are sufficient details of methods and analysis provided to allow replication by others?

Partly

Reviewer Expertise:

Bioethics, qualitative sociology, empirical ethics, African genomics, biobanking

We confirm that we have read this submission and believe that we have an appropriate level of expertise to confirm that it is of an acceptable scientific standard.

# Reviewer report: Reviewer response for version 1

**DOI:** 10.21956/wellcomeopenres.13658.r28289

**Published:** 2017-12-07

**Authors:** Malcolm de Roubaix, Ciara Staunton

**Affiliations:** 1 Department of Law, Middlesex University, London, UK; 2 Centre for Medical Ethics and Law, Department of Medicine, Faculty of Medicine and Health Sciences, Stellenbosch University, Cape Town, South Africa

I have the benefit of having read other reviewers' reports, and will limit my comments.

 This is an interesting paper and we would like to thank you for giving us the opportunity to review this paper. Work that aims to advance the cause of informed consent in the medical research in the rural African setting is to be welcomed. We appreciate the emphasis placed on context and contextual issues like the variety of languages. Overall however, there seems to be a disconnect between the introduction, results and discussion. The abstract and introduction speaks about the genetic research, yet the results and discussion appear to focus on medical research generally. More reflection on the use of the REA and its limitations should be incorporated into this paper.

 The following are specific comments on the paper.

**Introduction**

 Genomic research raises a number of challenges for the informed consent process and it is not clear why there is a focus on incidental findings only, particularly as the results speak to a wider range of issues. A broader discussion of these issues and, in particular, in the African setting would be good.

**Methods**

- The conceptual framework mentioned in the data analysis should be briefly discussed.
- Would it not also have been significant to interview traditional healers as well, since many Africans have to rely on their services, they are often trusted and revered members of rural communities and their views may be influential in those societies? Should they be involved in research at all, as has been done with HIV in many settings?

**Results**

- Under researcher/IRB members, does this mean that they are both researchers and IRB members?
- Throughout the results, it is often not clear whether you are referring to health research generally or podoconiosis. This should be clarified.
- At times the differences between researcher/IRB and other groups are discussed, but this is not consistent e.g. perceptions on informed consent for health research contains views of IRB/researchers only. These divergent views (if any) should be included. If consistent, this should also be made clear. Furthermore, were there any variations according to location?
- Under informed consent, you refer to it as a ‘tool’. Although one respondent does discuss it as a ‘tool’, it is a process. Moreover, in thinking of it only as a ‘tool’ presumably to inform’, one downplays the real issue, i.e., obtaining authentic consent. Informing is just one of the ingredients of informed consent. Being a process implies something developing over time, not instantaneously.
- Had the other groups any perceptions on informed consent as they were asked about it?
- Considering the questions asked about informed consent, was there any other than it being an important process that was discussed?
- In ‘decision making, recruitment and consent taking’, you refer to ‘most participants’ and ‘they’.  Again, here it is important to indicate which group you are referring to, and if there are differences.
- Factors affecting recruitment to research: would females not be comfortable being interviewed by any stranger, or just a male stranger? Spelling error on ‘sample type sought’.
- Previous research experience: does this mean that those with no previous exposure to research had sorcery concerns? Are some factors more important than others?
- The patient groups were not asked about incidental findings. Why is this?

**Discussion**

 The 4 ^th^ paragraph is confusing and raises a number of issues. You are stating that participants want to know basic information, but then state that information sheets and consent forms should have much more information. The discussion does not correlate with what has been stated in the results. How does this link to ‘genuine’ informed consent. Indeed, what is the difference between ‘genuine informed consent’ and ‘informed consent’. The term ‘authentic’ informed consent may be more appropriate.

 Incidental findings is a challenging topic and one that needs to be addressed in this context. Some reflection on the views expressed in these findings must be discussed. As the paper stands, the issuer regarding incidental findings is almost an afterthought, despite it appearing to be of importance in the introduction.

 Research should generally benefit the society/individuals who participate. How would this society benefit? How was feedback provided to the participants, if at all?

 Retention of samples/banking: This may also be an area of great concern. It is troubling that respondents may not have a clear understanding of what this means and the importance be down played, since it is ‘only’ buccal swabs we’re dealing with. What measures should be taken to improve this misunderstanding?

 Discussion on the REA, its use and limitations should be expanded.

 Those 20 page hardly comprehensible consent forms are problematic in all settings, more so in this context. Discuss methods of obviating this…e.g., a shortened say 2 page summary dealing with the main issues, in plain language.

Is the work clearly and accurately presented and does it cite the current literature?

Yes

If applicable, is the statistical analysis and its interpretation appropriate?

Yes

Are all the source data underlying the results available to ensure full reproducibility?

Yes

Is the study design appropriate and is the work technically sound?

Yes

Are the conclusions drawn adequately supported by the results?

Partly

Are sufficient details of methods and analysis provided to allow replication by others?

Partly

Reviewer Expertise:

NA

We confirm that we have read this submission and believe that we have an appropriate level of expertise to confirm that it is of an acceptable scientific standard, however we have significant reservations, as outlined above.

# Response

**Published:** 2026-02-19

**Authors:** Tewodros Gebresilase

**Affiliations:** Addis Ababa University/ Armauer Hansen Research Institute, Ethiopia

**Response to Reviewer 3 (Malcolm de Roubaix & Ciara Staunton)** **Introduction**

- Genomic research raises a number of challenges for the informed consent process and it is not clear why there is a focus on incidental findings only, particularly as the results speak to a wider range of issues. A broader discussion of these issues and, in particular, in the African setting would be good.
  - **Response: **We accept this suggestion. We have revised the Introduction to broaden the discussion of ethical challenges in genomic research beyond incidental findings. Specifically, we added text highlighting that data sharing, sample storage, and sample export are critical concerns in the African genomic landscape (citing Tindana et al., 2012). This broader context ensures the Introduction better reflects the wide range of issues covered in our Results, moving beyond a singular focus on incidental findings.

**Methods**

- The conceptual framework mentioned in the data analysis should be briefly discussed.
  - **Response**: We accept this suggestion. We have revised the "Data Analysis" section to include a brief summary of the conceptual framework so readers do not need to consult external references. We clarified that this framework systematically explores local perspectives across the continuum of the consent process, specifically focusing on: (i) perceptions of health research versus medical care, (ii) preferred channels for approaching and recruiting participants, (iii) individual versus communal decision-making practices, and (iv) preferences for information provision and consent documentation.
- Would it not also have been significant to interview traditional healers as well, since many Africans have to rely on their services, they are often trusted and revered members of rural communities and their views may be influential in those societies? Should they be involved in research at all, as has been done with HIV in many settings?
  - **Response**: We agree that a more critical reflection on the methodology is necessary. We have added a "Limitations" paragraph to the end of the Discussion section. In this section, we explicitly acknowledge the inherent limitations of a "rapid" assessment (typically 4–6 weeks) compared to long-term ethnography, as well as the potential for selection bias due to our reliance on community gatekeepers. Regarding the exclusion of traditional healers, we explain that while we recognize their influence in rural health-seeking behavior, our sampling was driven by the specific recruitment pathway of the genetic study (facilitated by health extension workers and clinic staff). Therefore, we prioritized stakeholders directly involved in that specific consent interface.

**Results**

- Under researcher/IRB members, does this mean that they are both researchers and IRB members?
  - **Response: **We have revised the "Study Design and Participants" section to explicitly clarify this definition. We state that this group consisted of "researchers who also served as Institutional Review Board (IRB) members." We interviewed these specific experts to capture perspectives that bridge both the scientific conduct of research and the ethical review process.
- Throughout the results, it is often not clear whether you are referring to health research generally or podoconiosis. This should be clarified.
  - **Response**: We have reviewed the Results section and added a clarifying statement to the opening paragraph. We explain that due to the research-naïve nature of the population, participants’ views on "perceptions of research" and "consent" tended to be about health research in general (often conflated with medical care). However, findings regarding specific logistics—such as recruitment channels, sample types (saliva), and explaining heredity—were directly focused on the context of the planned podoconiosis study.
- At times the differences between researcher/IRB and other groups are discussed, but this is not consistent e.g. perceptions on informed consent for health research contains views of IRB/researchers only. These divergent views (if any) should be included. If consistent, this should also be made clear. Furthermore, were there any variations according to location?
  - **Response**: We have revised the Results section to address these points. Regarding divergent views, we added text to the "Perceptions of informed consent and preparation of study documents" section explicitly contrasting researcher perspectives (who defined consent as an ethical "process") with community perspectives (who viewed it as a procedural step for receiving care). Regarding location, we inserted a statement at the beginning of the Results section clarifying that findings were consistent across both zones, with the exception of specific linguistic variations regarding the translation of technical terms.
- Under informed consent, you refer to it as a ‘tool’. Although one respondent does discuss it as a ‘tool’, it is a process. Moreover, in thinking of it only as a ‘tool’ presumably to inform’, one downplays the real issue, i.e., obtaining authentic consent. Informing is just one of the ingredients of informed consent. Being a process implies something developing over time, not instantaneously.
  - **Response:** We agree that informed consent is a dynamic process, not merely a tool. The reference to "tool" in the original text reflected the specific language used by interviewees when discussing the *information sheet*. We have revised the "Perceptions of informed consent" section to clearly distinguish these concepts. We now state that while stakeholders referred to the documentation as a communication "tool," they emphasized that consent itself is a fundamental ethical "process" of obtaining permission.
- Had the other groups any perceptions on informed consent as they were asked about it?
  - **Response: **In our analysis, we found that distinct perceptions of "informed consent" as a theoretical concept were largely unique to the Researcher/IRB group. As we have clarified in the revised "Perceptions of informed consent and preparation of study documents" section, patients and community members did not articulate a specific perception of "consent" as an isolated ethical stage. Instead, their views were embedded in their broader understanding of the relationship with health providers. When asked about consent, their responses focused on practical requirements (e.g., wanting to know the cause of the disease) and documentation preferences (e.g., the preference for written versus verbal agreement). We believe the current revision accurately reflects this distinction: professionals discussed the definition of consent, while community members discussed the conditions for agreement.
- Considering the questions asked about informed consent, was there any other than it being an important process that was discussed?
  - **Response: **Yes, our analysis extended well beyond defining consent as a process. As detailed in the revised "Perceptions of informed consent and preparation of study documents" and "Decision-making and recruitment factors" sections, the interviews covered:
    - **Content Preferences**: We found a clear divergence where community members prioritized practical information (disease cause and prevention) while researchers emphasized legalistic details (risks and contact info).
    - **Documentation Format**: We explored the debate between written consent (viewed by some as "tangible proof") versus verbal consent (preferred by those fearful of signing legal documents).
    - **Decision-Making**: We examined the tension between individual autonomy and family consultation, particularly when logistical burdens were involved.
- In ‘decision making, recruitment and consent taking’, you refer to ‘most participants’ and ‘they’.  Again, here it is important to indicate which group you are referring to, and if there are differences.
  - **Response: **We have revised this section (now titled "Decision-making and recruitment factors") to explicitly identify the stakeholder groups for each finding. We clarify that while decision-making was generally viewed as an individual act by patients (unless logistical burdens required family consultation), the specific barriers to recruitment (gender and sample type) were primarily identified by field workers. Additionally, regarding the findings on consent documentation (which we have moved to the earlier "Perceptions of informed consent" section for better flow), we now explicitly contrast the views of care coordinators (previously referred to as podoconiosis administrators), who preferred verbal consent, with those of patients, who preferred written consent. This revision removes all ambiguous references to "most participants".
- Factors affecting recruitment to research: would females not be comfortable being interviewed by any stranger, or just a male stranger? Spelling error on ‘sample type sought’.
  - **Response**: We have corrected the spelling of "sample type sought" in Table 2. Regarding the query on gender, our data specifically indicates that married women are uncomfortable being interviewed alone by male strangers. We have updated both the narrative text and Table 2 to explicitly specify "male stranger" rather than just "stranger" to ensure precision.
- Previous research experience: does this mean that those with no previous exposure to research had sorcery concerns? Are some factors more important than others?
  - **Response: **We have revised the "Decision-making and recruitment factors" section to address these queries. Regarding the relative importance of factors, we explicitly state in the text (and Discussion) that gender and sample type were identified by field workers as the most critical determinants of recruitment success. Regarding previous research experience, we confirm the reviewer’s interpretation: field workers observed that participants with *no* prior exposure to research were significantly more susceptible to rumors (such as fear of sorcery or blood-selling), whereas those with previous exposure—particularly through local treatment programs—did not exhibit these specific fears, as noted in Table 2.
- The patient groups were not asked about incidental findings. Why is this?
  - **Response: **We intentionally restricted the discussion of "incidental findings" to Researcher/IRB members because the concept was deemed too abstract for the study population. As our results indicate, many community participants already struggled to distinguish between "research" and "routine medical care" (therapeutic misconception). Introducing the concept of *secondary* genomic findings—unexpected information unrelated to the primary study—would have been difficult to translate and likely would have deepened the misconception that the study was a comprehensive health screening. We have updated the "Return of Incidental Findings" section to explicitly state that this topic was excluded from community interviews due to this complexity.

**Discussion**

- The 4 ^th^ paragraph is confusing and raises a number of issues. You are stating that participants want to know basic information, but then state that information sheets and consent forms should have much more information. The discussion does not correlate with what has been stated in the results. How does this link to ‘genuine’ informed consent. Indeed, what is the difference between ‘genuine informed consent’ and ‘informed consent’. The term ‘authentic’ informed consent may be more appropriate.
  - **Response: **We accept the comment regarding the terminology and have replaced "genuine informed consent" with "valid informed consent" throughout the manuscript to avoid tautology. Regarding the discussion on information provision, we have revised the text to resolve the tension between participants' preference for simple information and the ethical requirement for disclosure. We now clarify that our results support a "layered approach" to information provision: consent processes should begin with the practical medical information prioritized by the community (disease cause and benefits) as a bridge to explaining the abstract aims of genomic research. This approach directly links our results—specifically the use of local analogies—to our ethical recommendations.
- Incidental findings is a challenging topic and one that needs to be addressed in this context. Some reflection on the views expressed in these findings must be discussed. As the paper stands, the issuer regarding incidental findings is almost an afterthought, despite it appearing to be of importance in the introduction.
  - **Response: **We agree that the issue of Incidental Findings (IFs) required deeper reflection. We have expanded this section to explain that while international guidelines emphasize disclosure, our results suggest a specific context-dependent barrier: the risk of exacerbating therapeutic misconception. We have added an argument that introducing hypothetical secondary risks to participants who already struggle to distinguish research from clinical care may inadvertently confirm their belief that the study is a comprehensive health screening. Therefore, we conclude that in this specific setting, the immediate ethical priority is managing expectations about *primary* findings, rather than burdening the initial consent process with complex hypothetical scenarios.
- Research should generally benefit the society/individuals who participate. How would this society benefit? How was feedback provided to the participants, if at all?
  - **Response: **We have revised the Discussion to clarify the benefits provided. While monetary compensation was avoided to prevent undue influence, we followed the recommendation of local field workers to provide soap and sanitary materials to all participants. This provided a direct, tangible health benefit (promoting foot hygiene) relevant to the disease context. Regarding information sharing, we emphasize in the text that community engagement and sensitization were utilized not just for recruitment, but as critical mechanisms to build trust and provide study-related information to the community throughout the process.
- Retention of samples/banking: This may also be an area of great concern. It is troubling that respondents may not have a clear understanding of what this means and the importance be down played, since it is ‘only’ buccal swabs we’re dealing with. What measures should be taken to improve this misunderstanding?
  - **Response: **We agree that the non-invasive nature of saliva collection risks masking the significance of the biological data. In the revised Discussion, we explicitly address this by noting that the preference for saliva often stemmed from a misconception that it is "less significant" data than blood. To address this, we argue that the consent process must explicitly clarify that genetic data derived from saliva carries the same privacy and sensitivity implications as blood, ensuring participants understand that the resulting genomic data remains identical regardless of the collection method.
- Discussion on the REA, its use and limitations should be expanded.
  - **Response: **We have expanded the discussion on the REA methodology as suggested. Regarding its utility, we added text explicitly detailing how the REA allowed us to identify "invisible" barriers—specifically the linguistic conflation of research ( *Meremer*) with police investigation ( *Mermera*)—which standard ethical reviews might miss. Regarding limitations, we have added a new paragraph acknowledging that the "rapid" nature of the appraisal (4–6 weeks) necessitates a trade-off in anthropological depth compared to long-term ethnography. We also explicitly note the exclusion of traditional healers and the potential selection bias introduced by relying on government gatekeepers ( *Kebele* leaders), providing a more balanced view of the methodology’s constraints.
- Those 20 page hardly comprehensible consent forms are problematic in all settings, more so in this context. Discuss methods of obviating this…e.g., a shortened say 2 page summary dealing with the main issues, in plain language.
  - **Response: **We agree that lengthy, legalistic consent forms are particularly problematic in this context. In the revised Discussion, we address this by advocating for a "layered approach" to information provision. We argue that instead of a standard comprehensive form—which our findings suggest may signal a "lack of confidence" to the community—the process should begin with the practical medical information prioritized by the community (cause and prevention of podoconiosis). This acts as a bridge to explaining abstract genomic concepts using the local analogies identified in our results, effectively serving the function of a simplified summary.

# Reviewer report: Reviewer response for version 1

**DOI:** 10.21956/wellcomeopenres.13658.r26990

**Published:** 2017-11-22

**Authors:** Angela Fenwick

**Affiliations:** 1 Clinical Ethics and Law, Southampton General Hospital, University of Southampton, Southampton, UK

This paper describes a study using the Rapid Ethical Appraisal (REA) method to identify potential barriers to the consent process in preparation for a following genetic study on podoconiosis. The findings are potentially interesting but the paper needs some reworking before publication. 

 Title: The title does not reflect the contents of the paper and I would re-phase to indicate you used REA to identify the contextual factors/issues relevant for the podoconiosis study and to reflect the conclusion in your abstract. It currently looks like you are going to discuss the method of REA rather than apply it.   

 Abstract:

- The first sentence of your abstract is unclear and needs clarification: whose knowledge? And what do they need to know about research ethics to consent to a study?
- In the results section you appear to confuse the process of consent with evidence of consent which is repeated later in the paper. Do you mean that some participants preferred their consent to be recorded on a written consent form? If you clarify this in the body of your text you could come back to rephrase this sentence in the abstract. 
- You state that the ‘type of biological sample sought and gender’ were the two most important factors affecting the recruitment process’ but I assume you mean the two most important factors that the participants identified for the podoconiois study? These two are just listed in a table in your results section which seems inconsistent with the importance you attach to them.

 

 Introduction

- This is brief and some complex issues are dealt with rather superficially; for example in relation to therapeutic misconception: might the distinction between clinical care and research be less binary in some contexts, for example participating in research may give you a diagnosis (or a risk prediction) which you could then follow up. Similarly with autonomous decision making: to what extent do people really make decisions on their own? 
- I am not clear about the extent to which the use of REA is faster or cheaper than other ‘tools’ (which might be?) given the number of interviews and focus groups you conducted etc. Can you clarify? 

 

 Methods

- This needs expanding to include: some justification for your sample and sample size; how you decided whether to use a focus group or interview; how you dealt with these two different data sets during analysis. 
- You mention the prevalence of podoconiosis for over 15yrs but you only interviewed people over 18 so you need to give an explanation of why this was the case. 
- Under data analysis you say you used a conceptual framework described in previous studies and, whilst I don't think you need to go into lots of detail about this, I should not have to look up other studies to ascertain what you did. A brief summary would suffice.  

 

 Results

- In para 2 your themes should relate to your following headings more specifically. In general terms, this section would benefit from a re-organization. You move from present to past tense in this section on occasion: a general rule is that when you are reporting what participants felt, said etc you should use the past tense. 
- Perceptions of heath research: I am unclear whether you were asking participants about healthcare research in general or for your particular podoconiosis study: either way this needs clarifying. Without understanding this it is difficult to interpret your narrative (this is the case elsewhere also so a general clarification would help); for eg, the first quote indicates that research gives health status which may well be the case if a participant gets a diagnosis from their participation in research (as I indicated above), but this may not be the case in your particular study.  (See also reference to this in the 2 ^nd^ para of your discussion). The rest of this section reads rather clumsily with some repeated information and could do with streamlining. 
- Perceptions on informed consent for health research: this section is rather brief given the complexity of the concept. Could it be joined with another section? Is consent a ‘tool’? Isn’t it a process? The 2 ^nd^ quote relates to coercion and you could have highlighted this as it reflects more depth of thinking than your narrative indicates.  
- Preparation of consent form and information sheet: does the first sentence relate to the forms or to the wider process of consent which includes verbal explanation? This needs clarifying up-front - and in your second para - as from the quotes I would infer it was the latter, but this doesn't match your theme heading. The quote on confidentiality in particular seems to referring to issues inherent in the process rather than what they want included in information sheets.
- Approaching potential study participants: the title isn’t clear and isn’t clarified in your first sentence where ‘potential’ is not indicated. Are you referring to whom to approach first about a study (ie people who may not actually be the potential study participants but who act as gatekeepers/facilitators)?
- Information provision: the distinction between this theme and ‘Preparation of consent form and information sheet’ isn’t clear to me. Are you referring here to information about podoconiosis?
- Decision making, recruitment and consent taking: there is lots wrapped up in this heading and, as I indicated above, I think your table needs an accompanying narrative if it includes the 2 factors you consider most important. The first para is confusing and needs re-writing. In relation to the para that begins: ‘We found mixed views…’: I don't understand why this wouldn't be under the theme, ‘Preparation of consent form and information sheet’. 
- Return of incidental findings: this seems a huge topic to take on in such a brief way in this paper and I would suggest considering removing it as, as it stands, does not really tell me anything useful; and I have no idea whether the podoconiosis study would involve the return of IFs from what you have written. Furthermore I am not clear why you didn't ask patient and community members questions about Ifs. If you removed this theme you could expand on gender and sample type etc., if you are short of words.  

 

 Discussion

- In the first sentence you refer to ‘genuine informed consent’ which is a tautology. If consent is what you outline in your first paragraph then it doesn't need additional adjectives (you use it in your abstract also). In the 4 ^th^ para you say: ‘it is important to cover **all **aspects of the research to help participants reach an informed decision’, and ‘a **thorough** explanation’ is necessary but what does this mean in this context? How did your research help you understand what was appropriate? 
- If you re-organise your themes and take into account the issues raised above, then your discussion will be strengthened.
- Can you say more about what you wouldn't have known for the podoconiosis study if you had just relied on previous REA studies in Ethiopia? I would like a summary of what you took forward into the podoconiosis study. 

Is the work clearly and accurately presented and does it cite the current literature?

Partly

If applicable, is the statistical analysis and its interpretation appropriate?

Not applicable

Are all the source data underlying the results available to ensure full reproducibility?

Yes

Is the study design appropriate and is the work technically sound?

Yes

Are the conclusions drawn adequately supported by the results?

Partly

Are sufficient details of methods and analysis provided to allow replication by others?

Partly

Reviewer Expertise:

Ethical issues in genetic and genomic medicine; qualitative research methodology

I confirm that I have read this submission and believe that I have an appropriate level of expertise to confirm that it is of an acceptable scientific standard, however I have significant reservations, as outlined above.

# Response

**Published:** 2026-02-19

**Authors:** Tewodros Gebresilase

**Affiliations:** Addis Ababa University/ Armauer Hansen Research Institute, Ethiopia

**Response to Reviewer 2 (Angela Fenwick)** **Abstract:**

- The first sentence of your abstract is unclear and needs clarification: whose knowledge? And what do they need to know about research ethics to consent to a study?
  - **Response:** We accept this suggestion. We have revised the first sentence to clarify that the challenge arises from participants’ "limited familiarity with scientific research concepts," rather than a lack of knowledge about research ethics per se. Additionally, to address the concern regarding tautology, we have replaced "genuine informed consent" with "valid informed consent" throughout the abstract and manuscript.
- In the results section you appear to confuse the process of consent with evidence of consent which is repeated later in the paper. Do you mean that some participants preferred their consent to be recorded on a written consent form? If you clarify this in the body of your text you could come back to rephrase this sentence in the abstract. 
  - **Response:** We appreciate this distinction. We have rephrased the Abstract to clarify that the preference refers specifically to the documentation of consent. The text now specifies that while field workers preferred verbal consent to avoid literacy barriers, the majority of patients preferred "written consent (signature or fingerprint)" as tangible evidence of their agreement.
- You state that the ‘type of biological sample sought and gender’ were the two most important factors affecting the recruitment process’ but I assume you mean the two most important factors that the participants identified for the podoconiois study? These two are just listed in a table in your results section which seems inconsistent with the importance you attach to them.
  - **Response:** We accept this suggestion. We have revised the Abstract to explicitly attribute these factors to the field workers and care coordinators (previously referred to as podoconiosis administrators) who identified them as critical logistical barriers. Furthermore, to ensure the text is consistent with the importance placed on these factors in the Abstract, we have significantly expanded the Discussion section to analyze them in depth—specifically addressing the gender norms regarding private interviews and the misconception that saliva is "less significant" data than blood.

**Introduction**

- This is brief and some complex issues are dealt with rather superficially; for example in relation to therapeutic misconception: might the distinction between clinical care and research be less binary in some contexts, for example participating in research may give you a diagnosis (or a risk prediction) which you could then follow up. Similarly with autonomous decision making: to what extent do people really make decisions on their own? 
  - **Response**: We accept this suggestion. We have revised the Introduction to provide a deeper explanation of these nuances. Specifically, we added text acknowledging that in genomic research, the distinction between research and care is often blurred because the research itself provides medically relevant information (e.g., risk prediction), thereby offering potential clinical value. Regarding decision-making, we expanded the text to explicitly describe local practices as 'communal' or 'relational'—involving consultation with family heads and elders—rather than simply framing them as contrary to Western standards of autonomy.
- I am not clear about the extent to which the use of REA is faster or cheaper than other ‘tools’ (which might be?) given the number of interviews and focus groups you conducted etc. Can you clarify? 
  - **Response**: We have revised the Introduction to clarify the specific efficiency of the method. We specified that an REA cycle typically requires only 4–6 weeks and costs approximately ~2,250 USD per site. We explain that this distinguishes it from traditional, full-scale ethnographies or longitudinal qualitative studies, which often require months of fieldwork and significantly higher budgets.

**Methods**

- This needs expanding to include: some justification for your sample and sample size; how you decided whether to use a focus group or interview; how you dealt with these two different data sets during analysis. 
  - **Response**: We have expanded the *Methods* section to address these points:
    - **Sampling:** We clarified that purposive sampling was used to capture diverse stakeholder perspectives and that the final sample size was determined by data saturation.
    - **Method Choice:** We added a justification explaining that In-depth Interviews (IDIs) were used with patients to ensure privacy regarding sensitive topics, while Focus Group Discussions (FGDs) were used with community members to elicit shared social norms.
    - **Analysis:** We clarified that data from both sources were triangulated during analysis to compare individual narratives against group consensus.
- You mention the prevalence of podoconiosis for over 15yrs but you only interviewed people over 18 so you need to give an explanation of why this was the case. 
  - **Response: **The prevalence statistics cited (15+ years) refer to established epidemiological data. However, for this study on informed consent, we restricted recruitment to adults (≥18 years) as this is the legal age of majority for independent consent in Ethiopia. We believe the current description of participants as "adults" (18-68 years) in the *Results* sufficiently distinguishes our sample from the general prevalence data.
- Under data analysis you say you used a conceptual framework described in previous studies and, whilst I don't think you need to go into lots of detail about this, I should not have to look up other studies to ascertain what you did. A brief summary would suffice.
  - **Response: **We accept this suggestion. We have revised the *Data Analysis* section to include a brief summary of the conceptual framework. We clarified that this framework systematically explores local perspectives across the continuum of the consent process, specifically focusing on perceptions of research, recruitment strategies, decision-making dynamics, and consent preferences.

** Results**

- In para 2 your themes should relate to your following headings more specifically. In general terms, this section would benefit from a re-organization. You move from present to past tense in this section on occasion: a general rule is that when you are reporting what participants felt, said etc you should use the past tense. 
  - **Response: **We accept this suggestion. We have revised the introductory paragraph of the Results to ensure the listed themes correspond exactly to the subsequent section headings. Additionally, we have reviewed the entire Results section and standardized the verb tense, ensuring that all reports of participant views are consistent in the past tense (e.g., changing "use" to "used", "pose" to "posed").
- Perceptions of heath research: I am unclear whether you were asking participants about healthcare research in general or for your particular podoconiosis study: either way this needs clarifying. Without understanding this it is difficult to interpret your narrative (this is the case elsewhere also so a general clarification would help); for eg, the first quote indicates that research gives health status which may well be the case if a participant gets a diagnosis from their participation in research (as I indicated above), but this may not be the case in your particular study.  (See also reference to this in the 2 ^nd^ para of your discussion). The rest of this section reads rather clumsily with some repeated information and could do with streamlining. 
  - **Response**: We appreciate this feedback. We have revised the text to clarify that the interviews followed a "funnel" approach: starting with general perceptions of health research to assess baseline understanding, before moving to specific perceptions of the proposed podoconiosis study. To reflect this, we have restructured the section into two distinct subsections: "Perceptions on health research" (covering general views and therapeutic misconception) and "Specific perceptions regarding the podoconiosis study" (covering heredity and study design). We also added an introductory sentence explicitly outlining this structure.
- Perceptions on informed consent for health research: this section is rather brief given the complexity of the concept. Could it be joined with another section? Is consent a ‘tool’? Isn’t it a process? The 2 ^nd^ quote relates to coercion and you could have highlighted this as it reflects more depth of thinking than your narrative indicates.  
  - **Response**: We accept this suggestion. We have merged this section with the subsequent one, now titled "Perceptions of informed consent and preparation of study documents," to improve the narrative flow. We have also clarified that while participants often referred to the information sheet as a "tool," they explicitly recognized consent itself as a fundamental ethical "process." Furthermore, we added an interpretative sentence highlighting the participant’s insight that voluntariness is essential not just for ethics, but for the scientific validity of the data.
- Preparation of consent form and information sheet: does the first sentence relate to the forms or to the wider process of consent which includes verbal explanation? This needs clarifying up-front - and in your second para - as from the quotes I would infer it was the latter, but this doesn't match your theme heading. The quote on confidentiality in particular seems to referring to issues inherent in the process rather than what they want included in information sheets.
  - **Response**: We appreciate this insight. We have revised the text to clearly distinguish between the content requirements for written forms (which must be simple) and the challenges inherent in the verbal consent process. Regarding the quote on confidentiality, we agree it relates to the recruitment environment rather than the content of the forms. Accordingly, we have moved this quote to the " Approaching the community and potential study participants" section.
- Approaching potential study participants: the title isn’t clear and isn’t clarified in your first sentence where ‘potential’ is not indicated. Are you referring to whom to approach first about a study (ie people who may not actually be the potential study participants but who act as gatekeepers/facilitators)?
  - **Response:** We accept this suggestion. We have renamed the section “Approaching the community and potential study participants” to clearly distinguish between the gatekeepers and the study subjects. We also revised the opening sentence to explicitly state that the process involves “trusted community intermediaries” facilitating access to “potential study participants.”
- Information provision: the distinction between this theme and ‘Preparation of consent form and information sheet’ isn’t clear to me. Are you referring here to information about podoconiosis?
  - **Response:** We agree that the previous title ("Information provision") was ambiguous and appeared to overlap with the section on preparing consent forms. The distinction is that the previous section dealt with the *written* study documents, whereas this section focuses on *verbal* strategies (specifically analogies) used to explain abstract genetic concepts to participants with low literacy. To make this distinction explicit, we have renamed this section "Strategies for explaining complex genetic concepts." This clarifies that the focus is on pedagogical methods (such as the "improved seed" and "common cold" analogies) rather than general information about podoconiosis.
- Decision making, recruitment and consent taking: there is lots wrapped up in this heading and, as I indicated above, I think your table needs an accompanying narrative if it includes the 2 factors you consider most important. The first para is confusing and needs re-writing. In relation to the para that begins: ‘We found mixed views…’: I don't understand why this wouldn't be under the theme, ‘Preparation of consent form and information sheet’. 
  - **Response**: We have significantly restructured this section to address your concerns. We agree that the findings regarding verbal versus written consent fit more logically under the earlier theme, "Perceptions of informed consent and preparation of study documents," and have moved that paragraph accordingly to improve the flow. Regarding decision-making, we rewrote the opening paragraph to clearly distinguish between autonomous decision-making (common for low-burden studies) and family-based decision-making (required for studies involving travel, costs, or family planning). Finally, we added a specific narrative preceding Table 2 to explicitly highlight gender and sample type as the most critical recruitment factors identified by field workers
- Return of incidental findings: this seems a huge topic to take on in such a brief way in this paper and I would suggest considering removing it as, as it stands, does not really tell me anything useful; and I have no idea whether the podoconiosis study would involve the return of IFs from what you have written. Furthermore I am not clear why you didn't ask patient and community members questions about Ifs. If you removed this theme you could expand on gender and sample type etc., if you are short of words.  
  - **Response: **We acknowledge your concern regarding the scope of this section. However, we have chosen to retain it because it documents a critical ethical divergence between international guidelines ("duty to warn") and local realities ("right not to know" due to limited health infrastructure). regarding the absence of community views, we have revised the text to clarify that this exclusion was deliberate: introducing hypothetical secondary risks to participants who already struggle to distinguish research from clinical care ("therapeutic misconception") risked confirming their belief that the study was a comprehensive health screening. Finally, to address your suggestion about prioritizing recruitment factors, we have also significantly expanded the narrative regarding gender and sample type in the "Decision-making" section.

**Discussion**

- In the first sentence you refer to ‘genuine informed consent’ which is a tautology. If consent is what you outline in your first paragraph then it doesn't need additional adjectives (you use it in your abstract also). In the 4 ^th^ para you say: ‘it is important to cover all aspects of the research to help participants reach an informed decision’, and ‘a thorough explanation’ is necessary but what does this mean in this context? How did your research help you understand what was appropriate? 
  - **Response:** We accept the comment regarding the term "genuine informed consent" and have replaced it with "valid informed consent" throughout the manuscript to avoid tautology. Regarding the definition of "appropriate" explanation, we have revised the Discussion to resolve the tension between participants’ preference for simple information and the ethical requirement for disclosure. Our findings indicated that a standard "thorough" explanation—often interpreted as reading a lengthy, legalistic consent form—was counterproductive and signaled a "lack of confidence" to the community. Therefore, we have clarified that "appropriate" explanation in this context refers to a "layered approach": the consent process should begin with the practical medical information prioritized by the community (disease cause and prevention) as a bridge to explaining abstract genomic concepts using the local analogies identified in our results (e.g., "sowing seeds" for heredity).
- If you re-organise your themes and take into account the issues raised above, then your discussion will be strengthened.
  - **Response:** We accept this constructive suggestion. We have extensively revised and reorganized the Discussion section to directly align with the themes identified in our results. Specifically, the discussion now follows the logical progression of the consent process—addressing the tension between simple information and ethical disclosure, the specific role of gatekeepers, and the critical recruitment factors (gender and sample type). These structural changes have significantly strengthened the logical flow and ensured that every claim in the discussion is anchored in the data presented in the results.
- Can you say more about what you wouldn't have known for the podoconiosis study if you had just relied on previous REA studies in Ethiopia? I would like a summary of what you took forward into the podoconiosis study. 
  - **Response:** We have revised the Discussion to explicitly detail the unique findings of this REA that could not have been extrapolated from previous studies in Ethiopia. Specifically, without this context-specific assessment, we would not have known to implement three critical changes:
    - **Linguistic Context:** We identified a specific barrier where the Amharic term for research ( *Meremer*) was conflated with criminal investigation ( *Mermera*), necessitating the use of the term *Tinat* (study) to avoid fear of police involvement.

-
  -
    - **Gatekeepers:** Unlike previous findings where religious leaders were primary gatekeepers, our REA revealed that *Kebele* (local government) administrators were the essential authority figures needed to legitimize the study in these specific zones.
    - **Sample Misconceptions:** We found that the preference for saliva over blood often stemmed from a misconception that saliva was "less significant" data, prompting us to design a consent process that explicitly clarified that genetic data from saliva carries the same privacy risks as blood.

# Reviewer report: Reviewer response for version 1

**DOI:** 10.21956/wellcomeopenres.13658.r26871

**Published:** 2017-11-07

**Authors:** Jantina de Vries, Syntia Nchangwi Munung

**Affiliations:** 1 Department of Medicine, Faculty of Health Sciences, University of Cape Town, Cape Town, South Africa

**Overall**

 This is an interesting paper and we thank you for giving us the opportunity to review it and provide you with some feedback that may improve the manuscript. Our most important point is to challenge you to integrate in the Discussion section some critical feedback on the limits of the REA method, or at least some reflection on whether and when REAs should be conducted if similar other REAs have already been conducted on the same diseases and/or the same scientific methodologies, albeit in different populations. Specifically, we would challenge you to consider whether the REA you report in this study yielded new information that led to the design of a ‘better’ (more locally attuned) consent process than would have been designed without the REA having been conducted (drawing on the results of other published REA studies conducted in Ethiopia). Early in the manuscript, you hypothesise that the linguistic and cultural variation in this part of Ethiopia justified conducting another REA – did you find that hypothesis to be true (and please give concrete examples)? And if so, what is the implication for the design of future genomic studies?

 Below, we give you some feedback specifically on each of the sections of the manuscript. Some of these are mere suggestions that would enrich your discussion and description; others are more critical of the way you present your work. We hope that these suggestions are useful to you and look forward to the next iteration of this manuscript.

**Introduction**

- In paragraph 2 on Pg1 you refer to ‘therapeutic misconception’ and we would suggest that you add a reference to ‘diagnostic misconception’ there also. This term is now used in genomics research to describe the misconception by healthy controls that they are participating in a public screening endeavour;
- In paragraph 3 you mention one particular challenge to genomics research that has implications for the consent process – namely, incidental findings. Yet the design of genomics research harbours a number of features that impact on the consent process. In this paragraph, it would be good to reference one or two sources that outline the ways in which features of genomics research may impact on the consent process, and then also explain why in the Introduction you pick out the possibility for generating ‘incidental findings’ as a particular issue.
- We would suggest that the authors change their use of the term ‘developing countries’ to the now more commonly accepted ‘Lower and Middle Income’ countries or LMICs;
- You introduce the REA method as a ‘recent’ method (pg1) but it’s not altogether recent anymore. Perhaps you could rather say ‘one tool that has been applied successfully to ensure the adaptation of consent processes to local and cultural sensitivities is XYZ’.

 

**Study design and participants:**

- Add a sentence or two describing the group of “participants” referred to as Podoconiasis administrators. This is not very obvious compared to the other groups such as religious workers, researchers, IRB members;
- The FGDs were comprised of single-sex adults each containing 5 to 7 individuals. Maybe add a few sentences explaining why FGDs were composed of single-sex adults only and not otherwise.  

 

**Data Analysis**

- If the PI and co-investigators are authors of the paper, it may be better to use their initials as it has been done in the other cases.
- Under ‘data collection’ – were the interviews back-translated, or were the topic guides back-translated? And if so, what was the purpose of back-translating the topic guides by an independent person? (This is of course often done for informed consent to ensure the quality of the translation, but topic guides are a different kind of material and it’s not immediately obvious that these should also be ‘back-translated’ in the same way. It would be good to explain why you did that)

 

**Table 1**

 Maybe explain why there are differences in the methods used at the different sites. For example: Why were FGDs done with patients at East Gojjam and not at East Wellegam. This is important because  the authors argue that even though REAs have been previously used to explore ethical issues in Ethiopia, the problems identified might not have been be applicable to other Ethiopian populations that differ in social and community structure.

**Results**

- Overall, it may be good to get more of the researchers’ interpretation of the quotes. For example, what could explain why research participants associate health research with HIV testing and not testing for other diseases prevalent in that area?
- You describe in your results that people confused ‘research’ with police or criminal investigation, but you only offer one quote by an administrator who describes that risk exists. It is an important point though – it may impact on people’s voluntariness and ability to reject giving consent – and so merits being drawn out a bit. Have you got any quotes from prospective participants in the genomic study that illustrate this point? Could you perhaps give us a flavour of whether this was an important theme in your data or not?
- In your description of results, you sometimes describe all your interviewees as ‘participants’ and at other times you only talk about subgroups in your dataset (‘fieldworkers’ and ‘IRB members’). This is confusing, particularly when you shift focus in the article and it also only gives a very partial view of your data. E.g. when speaking about ‘understanding of research’ you talk about community members and patients, yet for ‘perceptions of IC’ you only talk about fieldworkers and IRB members – but this is not altogether clear when reading the paper. Perhaps you could talk about each stakeholder group for each of the themes you describe in the paper? 
- Were Researchers/IRB members the same group of people? Where they interviewed as researchers or as IRB members or both? This needs to be stated in the method.

 

**Perceptions on health research **

- The following quote “Research is used to increase new knowledge, whereas diagnosis is to find the disease.” (Patient 7, Gojjam) does not support the observation that patients did not understand the difference between research and clinical care. Rather, it confirms that the participant knew that diagnosis as part of routine care was different from research.
- How did the perception on health research by the different groups vary and what may be the contributing factors.

 

**Preparation of consent form and information sheet**

 The quote below may be moved to the next section. This is because it speaks more on approaching research participants than on the preparation of informed consent form and information sheet.

*“Confidentiality should be secured but it is a very difficult issue since most often many people are around…You may make an effort to keep people out of the hearing distance but in a village setting it is often not easy. Then the idea of confidentiality may also be a bit different because people have most of their private things in public. Is it not?" So, I think that is the most important thing, confidentiality.” (Researcher/IRB member 3).*

**Decision-making, recruitment and consent taking**

 The authors state that some of the “participants” also said they reached a decision on their own if the research benefits their health – further evidence that ‘therapeutic misconception’ is common in society.  A quote from a research participant (patient) is needed to support this statement.

**Discussion**

- A number of claims are made in the discussion section that have not been presented in the results section. The discussion should reflect the results presented and new concepts should ideally not be introduced in the discussion. For example:
  - Most participants residing in rural areas did not know the distinction between research and health care and thought they were being screened to receive treatment rather than participate in research. Maybe first describe the two study sites, e.g. whether they are rural or urban. In the results section, provide quotes that support the claim that those in rural areas did not support the distinction between research and health care;

- In your discussion, I don’t think it is appropriate to reference the Nigerian polio vaccine example to illustrate why CE is important in the context of health research – it is just one example and it’s from a completely different research context & country. There are stronger ethical grounds that make CE imperative and it would be more appropriate to draw on those.
- We find the 5 ^th^ paragraph of your discussion somewhat problematic. On the one hand, your data, as presented in the paper, seems to argue that participants do not want detailed information about the study but rather want information about the disease together with information about what will happen to their samples. Yet in this paragraph you argue that ‘participants should really know’ certain things and if they don’t understand then more work should be done to explain these things better. To us, this raises two questions, namely: 1) what was really in your data, and was there perhaps a range of views that lead you to describe the kinds of information participants should have and 2) how is the list that you describe informed by your data?

Is the work clearly and accurately presented and does it cite the current literature?

Yes

If applicable, is the statistical analysis and its interpretation appropriate?

Not applicable

Are all the source data underlying the results available to ensure full reproducibility?

Partly

Is the study design appropriate and is the work technically sound?

Yes

Are the conclusions drawn adequately supported by the results?

Partly

Are sufficient details of methods and analysis provided to allow replication by others?

Partly

Reviewer Expertise:

Bioethics, qualitative sociology, empirical ethics, African genomics, biobanking

We confirm that we have read this submission and believe that we have an appropriate level of expertise to confirm that it is of an acceptable scientific standard, however we have significant reservations, as outlined above.

# Response

**Published:** 2026-02-19

**Authors:** Tewodros Gebresilase

**Affiliations:** Addis Ababa University/ Armauer Hansen Research Institute, Ethiopia

**Response to Reviewer 1 (Jantina de Vries & Syntia Nchangwi Munung)** **Overall** **Introduction**

- In paragraph 2 on Pg1 you refer to ‘therapeutic misconception’ and we would suggest that you add a reference to ‘diagnostic misconception’ there also. This term is now used in genomics research to describe the misconception by healthy controls that they are participating in a public screening endeavour;
  - **Response**: We accept this suggestion. We have added the term "diagnostic misconception" to the Introduction and cited Masiye et al. (2017) to illustrate how it differs from therapeutic misconception, specifically regarding healthy controls who believe they are undergoing medical screening.
- In paragraph 3 you mention one particular challenge to genomics research that has implications for the consent process – namely, incidental findings. Yet the design of genomics research harbours a number of features that impact on the consent process. In this paragraph, it would be good to reference one or two sources that outline the ways in which features of genomics research may impact on the consent process, and then also explain why in the Introduction you pick out the possibility for generating ‘incidental findings’ as a particular issue.
  - **Response: **We accept this suggestion. We have revised the Introduction to explicitly acknowledge that genomic research impacts the consent process through various features, including data sharing, sample storage, and export complexities (citing Tindana et al., 2012). We further clarified that we singled out "incidental findings" because managing them is uniquely challenging in the African context due to limited healthcare infrastructure—a theme that directly connects to our study's findings regarding researcher/IRB views on the "duty to warn" versus the "right not to know."
- We would suggest that the authors change their use of the term ‘developing countries’ to the now more commonly accepted ‘Lower and Middle Income’ countries or LMICs;
  - **Response: **Accepted. We have replaced all instances of "developing countries" with "LMICs" or "low-resource settings" throughout the manuscript.
- You introduce the REA method as a ‘recent’ method (pg1) but it’s not altogether recent anymore. Perhaps you could rather say ‘one tool that has been applied successfully to ensure the adaptation of consent processes to local and cultural sensitivities is XYZ’.
  - **Response**: We agree that characterizing REA as a "recent" method is not accurate given the time that has passed since its initial development. We have revised the Introduction to describe it as "one tool that has been applied successfully" to tailor consent processes, and we removed the word "recently" from the subsequent paragraph regarding its assessment in Ethiopia.

**Study design and participants:**

- Add a sentence or two describing the group of “participants” referred to as Podoconiosis administrators. This is not very obvious compared to the other groups such as religious workers, researchers, IRB members;
  - **Response: **We have updated the terminology from "Podoconiosis administrators" to "care coordinators" throughout the manuscript to better reflect their role. We have also revised the "Study design and participants" section to explicitly define these individuals as staff members from the International Orthodox Christian Charities (IOCC) and Ethiopian Catholic Church (ECC) clinics who manage local podoconiosis treatment programs.
- The FGDs were comprised of single-sex adults each containing 5 to 7 individuals. Maybe add a few sentences explaining why FGDs were composed of single-sex adults only and not otherwise.  
  - **Response: **We have added a sentence explaining that single-sex FGDs were used to encourage open discussion, as mixed groups in this cultural setting often inhibit female participation. We have cited Abay et al. (2016) to support this methodological choice regarding gender dynamics.

**Data Analysis**

- If the PI and co-investigators are authors of the paper, it may be better to use their initials as it has been done in the other cases.
  - **Response: **We accept this suggestion. We have replaced the general terms "principal investigator" and "co-investigators" with the authors' initials (TTG, ZD, and GT) in the Data Analysis section to ensure consistency.
- Under ‘data collection’ – were the interviews back-translated, or were the topic guides back-translated? And if so, what was the purpose of back-translating the topic guides by an independent person? (This is of course often done for informed consent to ensure the quality of the translation, but topic guides are a different kind of material and it’s not immediately obvious that these should also be ‘back-translated’ in the same way. It would be good to explain why you did that)
  - **Response**: We have clarified in the *Data Collection* section that the topic guides were back-translated (rather than transcripts). We explain that this was done to ensure the conceptual equivalence of technical terms (e.g., "genetics", "randomization") across languages before data collection began, ensuring that the questions accurately conveyed the intended scientific concepts.

**Table 1:**

- Maybe explain why there are differences in the methods used at the different sites. For example: Why were FGDs done with patients at East Gojjam and not at East Wellega. This is important because  the authors argue that even though REAs have been previously used to explore ethical issues in Ethiopia, the problems identified might not have been be applicable to other Ethiopian populations that differ in social and community structure.
  - **Response: **We have added a clarifying sentence to the beginning of the Results section. We explain that the variation in methods (IDI vs. FGD) and participant numbers between sites was driven by the principle of data saturation. Additionally, the specific choice between IDIs and FGDs was guided by the logistical advice of local field workers regarding the feasibility and cultural appropriateness of convening groups in each specific context.

**Results**

- Overall, it may be good to get more of the researchers’ interpretation of the quotes. For example, what could explain why research participants associate health research with HIV testing and not testing for other diseases prevalent in that area?
  - **Response**: We have expanded the "Perceptions on health research" section to include our interpretation. We clarified that the confusion stems from the modality of the interaction: unlike routine clinical care which is self-initiated, research recruitment is externally initiated, mimicking the dynamic of mass HIV testing campaigns. We also referenced Negash et al. (2021) to support the finding that blood drawing often triggers specific fears of "blood selling" in this context.
- You describe in your results that people confused ‘research’ with police or criminal investigation, but you only offer one quote by an administrator who describes that risk exists. It is an important point though – it may impact on people’s voluntariness and ability to reject giving consent – and so merits being drawn out a bit. Have you got any quotes from prospective participants in the genomic study that illustrate this point? Could you perhaps give us a flavour of whether this was an important theme in your data or not?
  - **Response: **We appreciate this suggestion. We have added a quote from a Field Worker to the "Perceptions on health research" section that illustrates the tangible impact of this fear on voluntariness—specifically, the suspicion that signing forms relates to tax collection or property confiscation. We clarified that while prospective participants rarely voiced this fear directly to researchers (likely due to social desirability bias), it was a critical barrier consistently identified by community gatekeepers.
- In your description of results, you sometimes describe all your interviewees as ‘participants’ and at other times you only talk about subgroups in your dataset (‘fieldworkers’ and ‘IRB members’). This is confusing, particularly when you shift focus in the article and it also only gives a very partial view of your data. E.g. when speaking about ‘understanding of research’ you talk about community members and patients, yet for ‘perceptions of IC’ you only talk about fieldworkers and IRB members – but this is not altogether clear when reading the paper. Perhaps you could talk about each stakeholder group for each of the themes you describe in the paper? 
  - **Response**: We agree with the reviewer that using the generic term 'participants' was confusing. We have revised the *Results* section to explicitly distinguish between the views of different stakeholder groups. Specifically, we now contrast the views of researchers and field workers (who defined consent as a 'process') with those of patients (who focused on practical benefits), and we have specified which groups preferred verbal versus written consent to ensure the data is attributed to the correct source.
- Were Researchers/IRB members the same group of people? Where they interviewed as researchers or as IRB members or both? This needs to be stated in the method.
  - **Response:** We accept this suggestion. We have clarified in the *Study design and participants* section that this category consisted of researchers who *also* served as IRB members. We explain that we interviewed these individuals to capture dual perspectives: their practical experience in designing research and their regulatory experience in reviewing protocols.

**Perceptions on health research**

- The following quote “Research is used to increase new knowledge, whereas diagnosis is to find the disease.” (Patient 7, Gojjam) does not support the observation that patients did not understand the difference between research and clinical care. Rather, it confirms that the participant knew that diagnosis as part of routine care was different from research.
  - **Response: **We agree with the reviewer that this quote illustrates a correct understanding of research. We have restructured the *Perceptions on health research* section to present the findings more precisely: we first describe the majority view (therapeutic misconception) and then explicitly acknowledge the minority of participants, like Patient 7, who correctly distinguished research from clinical care.
- How did the perception on health research by the different groups vary and what may be the contributing factors.
  - **Response**: We have revised the *Results* section to explicitly articulate how perceptions varied by stakeholder group. We now contrast the views of patients (who largely perceived research as therapeutic aid or government investigation due to linguistic confusion), researchers (who viewed it through a scientific and ethical lens), and field workers (who focused on the practical logistics of community trust). We have also added text explaining the contributing factors, such as the linguistic conflation of "research" ( *Meremer*) with "police investigation" ( *Mermera*) and the resemblance of recruitment to mass HIV testing.

**Preparation of consent form and information sheet**

- The quote below may be moved to the next section. This is because it speaks more on approaching research participants than on the preparation of informed consent form and information sheet.
*“Confidentiality should be secured but it is a very difficult issue since most often many people are around…You may make an effort to keep people out of the hearing distance but in a village setting it is often not easy. Then the idea of confidentiality may also be a bit different because people have most of their private things in public. Is it not?" So, I think that is the most important thing, confidentiality.” (Researcher/IRB member 3).*
  - **Response: **We accept this suggestion. We agree that the quote regarding confidentiality in a "village setting" pertains to the environmental challenges of recruitment rather than the content of consent forms. Accordingly, we have moved this quote and its interpretation to the "Approaching the community and potential study participants" section.

**Decision-making, recruitment and consent taking**

- The authors state that some of the “participants” also said they reached a decision on their own if the research benefits their health – further evidence that ‘therapeutic misconception’ is common in society.  A quote from a research participant (patient) is needed to support this statement.
  - **Response**: We accept this suggestion. We have added a quote from a patient (Patient 9, Gojjam) to the "Decision-making and recruitment factors" section. This quote, which describes research as a "tool to maintain health," supports the finding that the willingness to decide autonomously is often driven by the therapeutic misconception of receiving direct medical benefit.

**Discussion** A number of claims are made in the discussion section that have not been presented in the results section. The discussion should reflect the results presented and new concepts should ideally not be introduced in the discussion. For example:

- Most participants residing in rural areas did not know the distinction between research and health care and thought they were being screened to receive treatment rather than participate in research. Maybe first describe the two study sites, e.g. whether they are rural or urban. In the results section, provide quotes that support the claim that those in rural areas did not support the distinction between research and health care;
  - **Response: **We accept this observation. As our study sites are predominantly rural (>90%), we have revised the Discussion to remove the implied comparison between rural and urban populations. The text now accurately reflects our results by stating that therapeutic misconception was a prevalent theme "across the study population," rather than implying a geographic divide.

- In your discussion, I don’t think it is appropriate to reference the Nigerian polio vaccine example to illustrate why CE is important in the context of health research – it is just one example and it’s from a completely different research context & country. There are stronger ethical grounds that make CE imperative and it would be more appropriate to draw on those.
  - **Response: **We accept this suggestion. We have removed the reference to the Nigerian polio vaccine boycott. We replaced it with specific findings from our own data regarding the influential role of *Kebele* administrators and Health Extension Workers, which provides a stronger, context-specific argument for the importance of community engagement.
- We find the 5 ^th^ paragraph of your discussion somewhat problematic. On the one hand, your data, as presented in the paper, seems to argue that participants do not want detailed information about the study but rather want information about the disease together with information about what will happen to their samples. Yet in this paragraph you argue that ‘participants should really know’ certain things and if they don’t understand then more work should be done to explain these things better. To us, this raises two questions, namely: 1) what was really in your data, and was there perhaps a range of views that lead you to describe the kinds of information participants should have and 2) how is the list that you describe informed by your data?
  - **Response: **We accept this constructive feedback. Our data revealed a clear divergence in views: while patients prioritized practical information (such as disease cause and prevention), researchers emphasized the ethical necessity of disclosing technical details. We have revised the Discussion to resolve this tension by proposing a "layered approach." We explain that the practical information requested by the community should be used as a "bridge" to facilitate comprehension of the necessary scientific concepts, rather than viewing these requirements as contradictory.
